# Retrieval practice improves memory in patients with schizophrenia: new perspectives for cognitive remediation

**DOI:** 10.1186/s12888-019-2341-y

**Published:** 2019-11-11

**Authors:** Camille Jantzi, Amaury C. Mengin, David Serfaty, Elisabeth Bacon, Julien Elowe, François Severac, Nicolas Meyer, Fabrice Berna, Pierre Vidailhet

**Affiliations:** 1 Université de Strasbourg, Faculté de Médecine, Strasbourg, France; 2 Hôpitaux Universitaires de Strasbourg, Service de Psychiatrie, 1 place de l’Hôpital, 67091 Strasbourg, France; 3 Hôpitaux Universitaires de Strasbourg, Département de Santé Publique, GMRC, Strasbourg, France; 4 Université de Strasbourg, Laboratoire de Biostatistique et Informatique Médicale, iCUBE UMR 7357, Illkirch, France; 5 Inserm U1114 – Neuropsychologie cognitive et Physiopathologie de la Schizophrénie, Strasbourg, France; 6 Fédération de Médecine Translationnelle de Strasbourg, Strasbourg, France

**Keywords:** Schizophrenia, retrieval practice, testing effect, memory, cognitive remediation

## Abstract

**Background:**

Schizophrenia is associated with severe cognitive deficits, particularly episodic memory deficits, that interfere with patients’ socio-professional functioning. Retrieval practice (also known as *testing effect*) is a well-established episodic memory strategy that involves taking an initial memory test on a previously learned material. Testing later produces robust long-term memory improvements in comparison to the restudy of the same material both in healthy subjects and in some clinical populations with memory deficits. While retrieval practice might represent a relevant cognitive remediation strategy in patients with schizophrenia, studies using optimal procedures to explore the benefits of retrieval practice in this population are still lacking. Therefore, the purpose of our study was to investigate the benefits of retrieval practice in patients with schizophrenia.

**Methods:**

Nineteen stabilised outpatients with schizophrenia (DSM-5 criteria) and 20 healthy controls first studied a list of 60 word-pairs (30 pairs with weak semantic association and 30 non associated pairs). Half the pairs were studied again (restudy condition), while only the first word of the pair was presented and the subject had to recall the second word for the other half (retrieval practice condition). The final memory test consisted in a cued-recall which took place 2 days later. Statistical analyses were performed using Bayesian methods.

**Results:**

Cognitive performances were globally altered in patients. However, in both groups, memory performances for word-pairs were significantly better after retrieval practice than after restudy (56.1% vs 35.7%, respectively, Pr(RP > RS) > 0.999), and when a weak semantic association was present (64.7% vs 27.1%, respectively; Pr(weak > no) > 0.999). Moreover, the positive effect of RP was observed in all patients but one.

**Conclusions:**

Our study is the first to demonstrate that retrieval practice efficiently improves episodic memory in comparison to restudy in patients with schizophrenia. This learning strategy should therefore be considered as a useful tool for cognitive remediation programs. In this perspective, future studies might explore retrieval practice using more ecological material.

## Background

Schizophrenia is associated with severe cognitive deficits, particularly episodic memory impairment. They interfere with patients’ socio-professional functioning [1, 2] and are correlated with occupational engagement, residential independence, and self-care [3]. Patients’ cognitive deficits can be improved by cognitive remediation therapies, being either restorative, compensatory or environmental, with immediate post-treatment effects on global cognition and durable effects on functioning [4, 5]. Studies have shown that cognitive remediation is more effective in association with a psychiatric rehabilitation program with a strategic approach [6]. Thus, understanding the kind of strategic approaches that are most effective in patients with schizophrenia is of major importance in building relevant or improving existing cognitive remediation programs. At present, most cognitive remediation programs in schizophrenia use either drill-and-practice or strategy-based techniques [5, 6]. While drill-and-practice entails repetitive practice of cognitive exercises that become gradually more difficult as performance improves, strategy-based techniques include a specific focus on strategies to be used. For instance, different strategies have been identified in the general population to improve memory through a semantic task—grouping items by category, visual repetition, counting words or letters, contextual encoding or sentence generation [7]. The use of these strategies varies with age [8–10], education and cognitive impairments [11–14]. In patients with schizophrenia, several studies have demonstrated a deficient use of encoding strategies that can be at least partially reversed when the efficient strategy used is explicitly provided to patients [15–18].

Retrieval practice (RP) is a well-established memory strategy that produces robust long-term episodic memory improvements in both healthy [19] and clinical populations with memory deficits (see Table 1 for a review of the results observed in clinical populations) [20–25]. It has been shown that taking a test on material has a greater positive effect on future retention than spending an equivalent amount of time restudying it: this effect is named the *testing effect* [26]. Roediger & Karpicke [26] described it in students by comparing the effect of repetitive testing (either taking a test in classrooms or self-testing by students) to repetitive study on the retention of various materials (e.g., word lists, prose passages) and with various modes of final recall (e.g., free recall, cued recall, multiple-choice). Their results convincingly demonstrated that testing is a relevant method to improve learning. Rowland et al. [19] later demonstrated that some parameters fostered testing effect, such as the material to be learnt (prose or paired associates), the kind of initial test (cued or free recall), the presence or absence of feedback during the initial test, and the retention interval (1 day or more). Studies on clinical populations with memory deficits were mostly conducted by Sumowski and collaborators [20–25]. The tests comprised weakly associated word pairs as a stimulus, a cued-recall method at both the initial and final tests, feedback during the initial test, and both short- and long-retention intervals (from 30 min to 30 days). They confirmed that RP improved memory performance in final recall in patients with multiple sclerosis, traumatic brain injury or HIV. The authors highlighted that RP could be effective for future rehabilitation methods.

**Table 1:** Retrieval practice on memory-impaired clinical populations: previous results

First author(s) (Year)	Population & participants	Study design	Main results
Avci et al. **(**2017**)**	52 people living with HIV21 seronegative controls	3 (learning condition: RP, MR, SR)	Large main effect of learning condition where participants recalled significantly more VPAs studied through RP compared with MR and SR.
Coyne et al. **(**2015**)**	15 pediatric survivors of Traumatic Brain Injury (TBI) aged 8 to 16 years with below-average memory.	2 (stimulus type: VPA, FNP)*3 (learning condition: RP, MR, SR)	Very large main effect of learning condition on delayed recall. RP led to better memory than did both MR and SR in all subjects, and SR better memory than MR.
Sumowski, Chiaravalloti et al. (2010)	32 persons with Multiple Sclerosis (MS)16 demographically matched healthy controls	3 (learning condition: RP, MR, SR)	Very large main effect of learning condition on VPA delayed cued recall. In MS patients, large mnemonic advantages for VPAs learned through RP relative to SR, SR relative to MR, and RP relative to MR. RP was the best learning condition for 90% of all participants (MS: 91%; HC: 88%).
Sumowski, Coyne et al. (2014)	10 memory-impaired survivors of severe TBI	3 (learning condition: RP, MR, SR)	Large main effect of learning condition after the short delay. Enduring beneficial effect of RP: the large effect of learning condition remained after the long delay.
Sumowski, Leavitt et al. (2013)	12 memory-impaired MS patients	3 (learning condition: RP, MR, SR)	Large main effect of learning condition after the short delay. Enduring beneficial effect of RP: the large effect of learning condition remained after the long delay.
Sumowski, Wood et al. **(**2010**)**	14 persons with chronic memory impairment following a TBI 14 age-matched healthy controls	3 (learning condition: RP, MR, SR)	Large effect of learning condition on delayed cued-recall in both groups, with RP leading to the best recall, followed by SR, and then MR. RP was the best strategy for 93% of persons with TBI.

*Note:* In RP and SR, material was presented in a spaced fashion. *Abbreviations: RP* Retrieval Practice, *MR* Massed Restudy, *SR* Spaced Restudy, *VPA* Verbal Paired Associates, *FNP* Face-Name Pairs, *TBI* Traumatic Brain Injury, *MS* Multiple Sclerosis

To our knowledge, no study has ever investigated testing effect in patients with schizophrenia to evaluate its relevance for cognitive remediation. However, previous studies in schizophrenia have explored retrieval practice using a different procedure called retrieval-induced forgetting (RIF). This paradigm refers to the fact that, under particular conditions, episodic memory recall can suppress the accessibility of semantically related information [27–30]. Thereby, Nestor et al. showed that learning words through RP (RP+) induced the forgetting of other related words (i.e., belonging to the same category) for which RP was not practiced (RP-), in comparison to unpracticed words from another category (NRP) [28]. Their results confirmed that (1) RP improves recall at the final test in comparison to unpracticed material (RP- and NRP conditions), and that (2) the inhibitory mechanism leading to the loss of retrieval access to the unpracticed related items is preserved in patients with schizophrenia. However, the procedure used to assess RIF differs in several points from those used to assess testing effect: first, it does not compare RP to another learning condition, such as restudy; secondly, the retention interval is quite short (5–20 min), which may reduce the efficacy of RP on episodic memory (see Table 2).

**Table 2:** Retrieval practice in schizophrenia: previous results

First author(s) (Year)	Population & participants	Study design	Main results
AhnAllen et al. (2007)	18 right-handed male patients with schizophrenia Mean age = 42.59 years, Mean length of illness = 18.5 years, Mean chlorpromazine equivalent = 422.53 mg 18 right-handed male healthy controls	RIF: 2 (associative strength: strong, weak)*3 (learning condition: RP+, RP-, NRP)	Patients with schizophrenia recalled fewer category-exemplar pairs (M = 33.92, SD = 15.66) than did normal controls (M = 50.48, SD = 14.41). Significant effect for group (ANCOVA), F (1, 34) = 12.64, *p* = .001, item, F (2, 33) = 35.42, *p* < .001, and associative strength, F (1, 34) = 30.86, *p* < .001.
Allen et al. **(**2003**)**	10 patients with schizophrenia 10 HC	2 (associative strength: strong, weak)*3 (learning condition: RP+, RP-, NRP)	Control subjects displayed similar patterns of retrieval practice (RP+ > NRP > RP-) for both strong and weak categories, though schizophrenic subjects showed evidence of a disproportionate drop in recall for RP+ weak categories-exemplar pairs in relation to their close to normal recall for RP+ strong categories-exemplar pairs
Nestor et al. **(**2005)	17 right-handed male patients with schizophrenia Mean age = 45.53 years, Mean length of illness = 21.27 years, Mean chlorpromazine equivalent = 331 mg 18 right-handed male HC	RIF: 3 (learning condition: RP+, RP-, NRP)	The control group had mean recall rates of 72.78% (SD = 18.98) for RP+ items, 39.52% (SD = 20.30) for RP- items, and 46.84% (SD = 12.61) for NRP items. The patient group had mean recall rates of 49.37% (SD = 19.98) for RP+ items, 21.41% (SD = 19.40) for RP- items, and 27.68% (SD = 11.42) for NRP items. ANCOVA yielded a significant effect for group, F = 11.038, (1, 28), *p* < 0.01 For the initial category-word stem completion task of retrieval practice, controls completed 86.9% (SD = 12.71) of the stems versus 75.89% (SD = 16.49) for patients with schizophrenia.
Soriano et al. (2010)	30 outpatients with schizophrenia Gender (m/f) = 26/4 Mean age = 39.17 years, Mean length of illness = 16.3 years, Mean chlorpromazine equivalent unknown. 18 HC, Gender (m/f) = 8/12	RIF: 3 (learning condition: RP+, RP-, NRP)	The facilitation effect of practice was significant for patients, F(1.29) = 68.15, MSE = 11,179.45, *p* < .05, ω^2^ = .7 with participants recalling more RP+ than NRP items. The percentage of correct recall in the retrieval practice phase was 73.9 for the schizophrenic group, and 75.1 for the control group.

*Note:* In RP and SR, material was presented in a spaced fashion. *Abbreviations: HC* Healthy Controls, *RP+* Retrieval Practice, *RP*-; No Retrieval Practice with category-exemplar pairs of the same category than in RP+ condition. NRP; No Retrieval Practice with category-exemplar pairs of another category than in RP+ condition

In fact, studies on RP in healthy subjects produced larger testing effects when the retention interval was at least over 1 day [19]. Consequently, studies implementing optimal procedures to verify that RP yields noteworthy memory improvements in patients with schizophrenia are still lacking. Our study is the first to compare RP to restudy in schizophrenia. Because patients with schizophrenia show an impaired ability to spontaneously use effortful encoding strategies but have a tendency to use rote repetition to learn new information such as weakly associated word pairs [31], RP may help them to engage in an efficient encoding strategy and improve their episodic memory performances. In these circumstances, we hypothesise that RP will efficiently improve episodic memory performances in patients with schizophrenia and then represent a relevant tool for cognitive remediation.

## Methods

### Participants

Twenty patients with schizophrenia (DSM-5 criteria) [32] and twenty healthy controls were included. The 2 groups did not differ in terms of age, gender and years of schooling. Patients were recruited at the University Hospital of Strasbourg, Psychiatry Department. Patients included were clinically stabilized outpatients and their medication had remained unchanged for the last 2 months. All patients but one were taking antipsychotic medications with a mean daily dose of 290 mg/d of chlorpromazine equivalent (CpzEq). Two patients (10%) were taking first-generation antipsychotics, and 17 (85%) second-generation antipsychotics. Two patients (10%) were taking benzodiazepines with a mean dosage of 0.5 mg/d of lorazepam equivalent. Four patients (20%) were taking antidepressants for more than one year (SSRIs or SNRIs). None of them were taking antiparkinsonian medication. Mean duration of illness was 17 years (*SD* = 11.3; range = 4 to 39). Symptoms of schizophrenia and depression were assessed using the Positive and Negative Syndrome Scale (PANSS) [33] and the Calgary Depression Scale (CDSS) [34], respectively. One patient was excluded afterwards due to clinical depression (CDSS = 13). Controls were recruited by billposting and through a healthy volunteer register. Every participant was of French native language. Exclusion criteria were: medical history of neurological disorder, current substance use disorder and current benzodiazepine treatment superior or equivalent to 1 mg/d of lorazepam and depression. Controls had neither personal nor family history of psychiatric disorder.

### Procedure

#### Neuropsychological evaluation

In the first session, every subject went through a neuropsychological evaluation. Pre-morbid IQ was assessed using the French validated version of the National Adult Reading Test (fNART) [35]. Episodic verbal memory was evaluated using the logical stories test of the Wechsler Memory Test, third edition (WMS-III) with a first recall and a differed recall separated by an intercurrent task, allowing to assess memory retention [36]. Processing speed was assessed using the codes & symbols test of the Wechsler Adult Intelligence Scale, fourth edition (WAIS-IV) [37]. Short-term memory and working memory were evaluated by the direct and reverse digit span subtest of WAIS-IV, respectively. The Mill Hill test assessed the ability to recall learned information and verbal communication. It estimates the level of vocabulary, that is, memory storage and retrieval of verbal knowledge; it comprises 2 subtests: word definitions and synonyms selection [38]. Executive functioning was evaluated using the Trail-Making Test [39] and both phonologic and semantic fluency tasks [40]. In these latter tests, the participants had 2 min to give as many words as they could starting with the letter “r” (phonological fluency), and 2 min to give as many fruit names as possible (semantic fluency). Selective visual attention was evaluated through Ruff 2 & 7, where participants had to cross “2” and “7” among other items [41].

#### Testing effect protocol

The second session took place 1 to 15 days later. It was dedicated to learning and relearning. Items to be learned consisted of 60 word-pairs, half with a weak semantic association (e.g., sugar-tea) and half with no association (e.g., bone-lemonade). Items were chosen from a French database of word association norms established for 366 concrete object names [42]. Pairs with and without semantic association did not differ in terms of occurrence frequency in French language (*Pr*(weak>no) = 0.926) and amount of letters (*Pr*(weak>no) = 0.291). Participants first learned the 60 pairs of words: each pair was projected on a computer screen for 8 s and participants were asked to learn the word pairs and to tell them aloud. Relearning was immediately conducted (in a different order than during learning) within 2 different conditions: retrieval practice (RP) for half the pairs and restudy (RS) for the other half. In the RS condition, word pairs were presented again for 12 s, with the same instructions as in the study phase. In the RP condition, only the first word of each pair was shown for 10 s, and participants had to recall aloud the second word of the pair before the entire pair was shown again for 2 s (feedback). In each condition, word pairs were presented 3 times: once for learning and twice for relearning. The 60 pairs of words were pseudorandomized so that presentation and representation of a same pair were separated one from another by at least 3 other pairs (spaced RP and RS) [22]. Pairs of words and strength of association were counterbalanced: we generated four different versions of the material so that the order of presentation of word pairs (either weakly or non-associated) and their assignation to either RP or RS condition were different for each version. Therefore, a fourth of the participants received each of the versions. The total duration of the learning/relearning session was of 32 min.

**Fig. 1:**
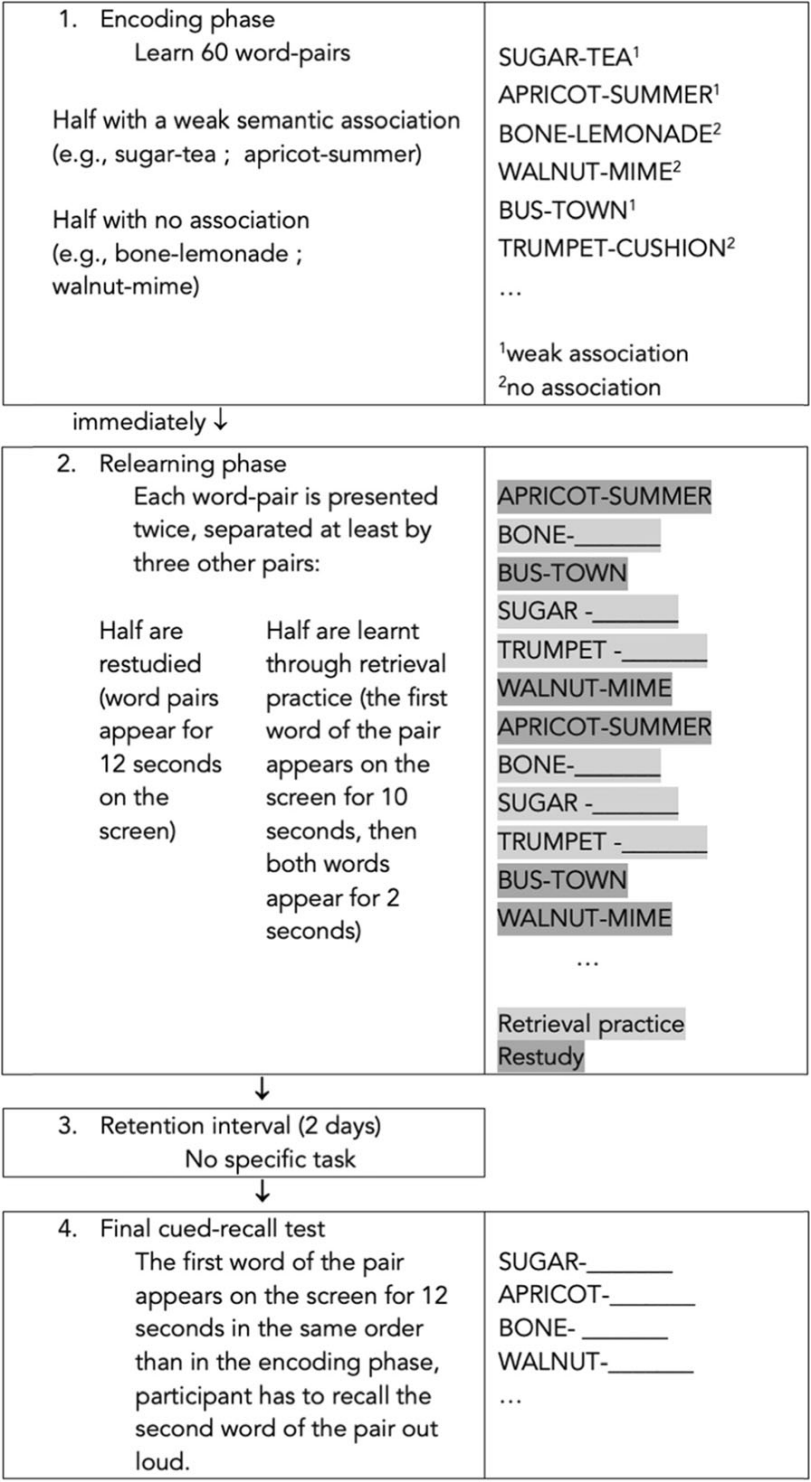
Experimental procedure for exploring the testing effect. Illustration of the 4 phases of the experimental procedure employed to explore the testing effect in patients with schizophrenia and controls

The third session consisted in the final memory test and took place 2 days after the learning/relearning phase. The first word of every 60 pairs was presented on a computer screen for 12 s each and the participant had to recall the second word of the pair aloud. The experimenter noted the answers. Pairs were presented in the same random order than in the encoding phase. The procedure is illustrated in Fig. 1.

#### Statistical analyses

Statistical analyses were performed using Bayesian methods [43] as it is more and more recommended in science in general and in experimental psychology in particular.^1^ In addition to discarding criticized *p*-values, Bayesian statistics provide a distribution of the probability that (in our case) the performance of patients is lower than that of controls (which is not to confound with the null hypothesis testing of classical statistics, although *p*-values are often misleadingly interpreted as reflecting this probability) [44, 45]. Moreover, Bayesian statistics offer the advantage to include prior knowledge in the analyses and to test the robustness of the results using sensitivity analyses. They test whether non-informative priors (a purely theoretical condition in which the researcher has no prior knowledge on a phenomenon to test - this purely theoretical condition is however the basic assumption underlying traditional frequentist statistics) or even pessimistic priors (priors that challenge authors’ hypotheses) influence the results of this abovementioned probability.

Sociodemographic variables were compared between groups using univariate linear or logistic regression analyses and cognitive variables with Beta regression analyses [46].

The number of word-pairs recalled during the initial memory test was compared between groups with a Beta regression given that the number of word pairs was not symmetrically distributed and was bounded (between 0 and 60).

The number of word-pairs recalled during the final memory test in each 4 situations (weak association – RS; weak association – RP; no association – RS; no association - RP) was treated as repeated measures. Therefore, a mixed model was used which took the intra-subject variance into account. For similar abovementioned reasons, a Beta regression and predictor variables included condition (RP vs. RS), semantic association (no vs. weak) and group (patients vs. controls). The influence of each predictor was examined first in separate univariate analyses. Interactions between predictors were secondarily examined in multivariate analyses including all predictors. The probability related to each factor is written *Pr*(RP > RS) (i.e., the probability that the number of pairs retrieved in RP condition is higher than that of pairs retrieved in RS) and is written *Pr*(OR > 1) for interactions. We considered both large values (i.e., > .95) and small values (i.e., < .05) of *Pr* as reflecting meaningful effects of the factor under consideration, given that *Pr*(RP > RS) = 0.95 is equivalent to *Pr*(RS > RP) = 0.05.

Based on previous data by Akdogan et al., we used informative priors for condition and semantic association for the univariate analyses [47]. If Theta is the coefficient of the predictor variable in the Beta regression, the normal distribution *N* [M +/− SD] for Theta was *N* [0.556, 0.499] for condition and *N* [1.214, 0.462] for semantic association (see details in Additional file 1: Table S1). It amounts to expecting an OR equal to 3.37 with a 95% credible interval (CI) of 1.90 to 4.84 for condition and OR = 1.74 [− 1.03 – 4.52] for semantic association. A non-informative prior was used for the group considering that this study was first to investigate RP in its present form in patients with schizophrenia.

## Results

Cognitive performances were significantly lower in patients than in controls in almost all neuropsychological tests (except for phonological fluency) (see Table 3).

**Table 3:** Sociodemographic and general cognitive performance (z scores) of patients with schizophrenia and controls

	Controls (*n* = 20)		Patients with schizophrenia (*n* = 19)		Statistics				
					Theta		*CI* 95%		*Pr*(Theta > 0)
	*M*	*SD*	*M*	*SD*	*M*	*SD*	2.5%	97.5%	
Socio-demographic variables									
Age	37.4	10.2	38.5	10.0	−.114	.337	−.777	.550	.365
Gender (number of men, %)	13	65.0	13	68.4	.136	.641	−1.118	1.402	.584
Years of schooling	12.6	1.7	11.9	2.1	.341	.331	−.311	.998	.852
Cognitive variables									
fNART	104.2	6.3	97.9	8.8	.770	.325	.129	1.408	.990
WMS III first recall	.748	.715	−.334	1.021	.678	.184	.314	1.039	>.999
differed recall	.932	.849	−.112	1.028	.681	.205	.277	1.084	>.999
total recall	.982	.577	−.298	.987	.836	.178	.484	1.186	>.999
WAIS IV symbols	−.282	.925	−1.165	.909	.508	.175	.165	.853	.998
code	−.333	.771	−1.498	1.044	.716	.193	.337	1.097	>.999
letter-number sequence	.633	1.097	−.389	1.205	.597	.226	.151	1.042	.995
total number memory	.549	.898	−.446	.982	.664	.205	.259	1.068	.999
Working memory direct span	−.557	1.031	−.988	1.010	.265	.206	−.144	.671	.902
indirect span	−.164	.857	−.761	.880	.435	.203	.033	.831	.983
Fluency phonological	.266	.922	.301	1.079	−.016	.180	−.373	.339	.463
semantic	.281	1.101	−.301	.931	.581	.291	.004	1.150	.976
Mill Hill	−.618	1.097	−.947	1.495	.150	.227	−.297	.596	.995
TMT A (motor speed)	.032	.883	−.944	1.274	.588	.223	.147	1.024	.995
(letter-number sequence)	.532	.622	−.484	1.455	.628	.231	.169	1.081	.996
(flexibility index)	.462	.448	.091	.587	.365	.176	.016	.709	.980
Ruff 2 & 7 total speed	.885	1.285	−.206	1.051	.678	.246	.191	1.162	.996
total accuracy	.725	.365	.383	.824	.278	.181	−.081	.634	.938
Clinical variables									
PANSS total			52.3	16.7					
positive			12.0	6.5					
negative			15.3	5.3					
CDSS			1.4	2.8					
Chlorpromazine equivalents (mg)			290						

*Note*: Results are presented as Theta with a 95% Credible Interval (CI), with the probability of the Theta being above 0: *Pr*(Theta> 0). A large *Pr*(Theta> 0) value (e.g., > 0.95, > 0.975, or 0.99) must be interpreted as indicating lower values for patients compared to controls (for predictor group). A small value of *Pr*(Theta> 0), for instance, < 0.05, 0.025, or 0.01, reflects higher values for patients compared to controls. It is worth noting that the probability *Pr*(Theta> 0) can be interpreted as 1 – *Pr*(Theta< 0). Thus, probability values near 1 and 0 both indicate a significant effect

*PANSS* Positive and Negative Syndrome Scale, *CDSS* Calgary Depression Scale for Schizophrenia, *WCS* Wechsler Cognitive Scale, *WAIS* Wechsler Adult Intelligence Scale, *TMT* Trail Making Test, *fNART* French National Adult Reading Scale

Regarding the initial test, the number of pairs recalled directly after the learning phase (considered as baseline performance) were lower in patients than in controls (43.4 and 56.5% respectively, *Pr*(patients > controls) = 0.008). However, the comparison between baseline scores and scores at the final test indicated that the improvement of performance observed (improvement of 3.6 and 7.3% of word-pairs recalled at the final test respectively, OR = 1.076, CI95%:1.026 – 1.128; *Pr*(final > baseline) = 0.998)^2^ was comparable in both groups (the interaction between the group and time (baseline test vs. final test) was not relevant (*Pr* = 0.179). Nevertheless, despite this improvement, patients did not reach the “baseline performance” level of controls during the final test.

**Fig. 2:**
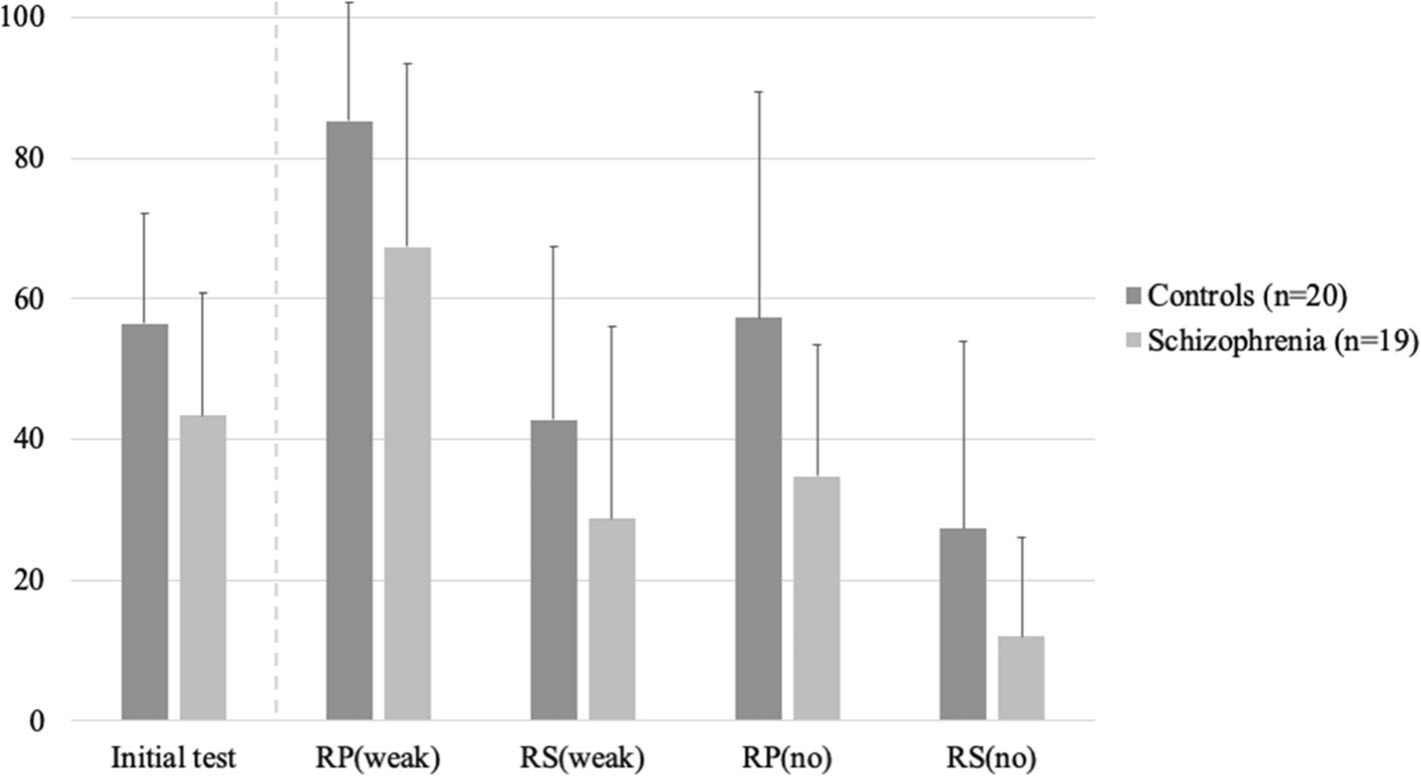
Percentage of words retrieved at initial and final cued-recall. Description of data: Comparison of the percentage of words retrieved by patients with schizophrenia and controls at initial test (i.e. in the Retrieval Practice condition only), and at final test in 4 different conditions: RP(weak); Retrieval Practice with weakly-associated word-pairs. RS(weak); Restudy with weakly-associated word-pairs. RP(no); Retrieval Practice with non-associated word-pairs. RS(no); Restudy with non-associated word-pairs. Legend: RP; Retrieval Practice. RS; Restudy

Univariate analyses showed that word-pairs were better recalled through RP than through RS (56.1% vs 35.7%, respectively; OR = 2.29, CI95%:1.67 – 3.12, *Pr*(RP > RS) > 0.999) and when a weak semantic association was present (64.7% vs 27.1%, respectively; OR = 5.45, CI95%:4.12 – 7.15, *Pr*(weak > no) > 0.999). Patients’ performances were lower than controls’ performances (37.4% vs 53.1%, respectively; OR = 0.56, CI95%:0.33 – 0.93, *Pr*(patients>controls) = 0.014) both in the weak and in the no association conditions (see Fig. 2 or Additional file 1: Table S2).

Multivariate analyses confirmed the relevant effects of group, condition and association and showed that RP was significantly more effective for word-pairs with weak semantic association as reflected by a relevant interaction between condition and semantic association (*Pr* = 0.998). In contrast, neither the interactions between group and condition or semantic association nor the interaction between all predictors were relevant (all *Pr* between 0.2 and 0.9) (see Additional file 1: Table S3). Importantly, the visual inspection of individual data indicated that all patients but one presented with a testing effect. For readers who are more familiar with frequentist statistic, results and effect sizes are reported in Additional file 1: Table S4. They indicate that all main effects and interactions were also significant using classical frequentist statistical analyses.

Sensitivity analyses were performed firstly using non-informative priors and secondly using pessimistic priors (i.e. priors in the opposite direction of the expected effects). The estimated coefficients remained unchanged (see Additional file 1: Table S5), suggesting that the estimations of coefficients were mostly driven by the data we collected and not by the expected results.

In order to investigate whether baseline cognition or performance at the initial test (baseline performance) had an impact on RP, we performed complementary analyses in which each of the following cognitive measures (fNART, Mill-Hill, WMS III total recall, working memory indirect span, semantic fluency, Ruff speed and accuracy) was added separately and successively to the initial Beta regression analysis. In all cases, main effects of condition and semantic link as well as all interactions remained unchanged. However, although the initial effect of group (*Pr*(patients > controls) = 0.03) was not influenced by the number of years of schooling, Mill Hill score, Ruff accuracy, and semantic fluency, this effect was smaller when working memory and Ruff speed were taken into account (*Pr*(patients > controls)s = 0.07), and disappeared when fNART or baseline performance were taken into account (*Pr*(patients > controls) = 0.25 and *Pr*(patients > controls) = 0.28, respectively).

## Discussion

Our study showed that retrieval practice can better improve long-term episodic memory performance than restudy in patients with schizophrenia, and that this so-called testing effect is similar to that reported in control participants. To our knowledge, only six previous studies demonstrated that retrieval practice improves memorisation in clinical populations with cognitive difficulties (traumatic brain injury, multiple sclerosis and HIV), while no study has shown its superiority to restudy in patients with schizophrenia. Moreover, all but one patient benefitted from RP in our study, indicating that this method should be helpful in a large proportion of patients with schizophrenia.

Although our results are very encouraging, they raise several methodological issues. First, as procedural similarities exist between the initial and final testing, one may argue that improvement of cognitive performances with RP only rely on a practice effect, that is, the increase in test scores that occurs upon repeating neuropsychological testing. To highlight practice effect, Goldberg et al. (2007) compared the test scores at t0 and t1 in patients with first-episode schizophrenia receiving antipsychotic medication to those of healthy controls (HC) [48]. They concluded that cognitive enhancement between t0 and t1 resulted mostly from practice effect, as the effect sizes for performance improvements were generally equal in patients and HC. In parallel, the *transfer appropriate processing theory* of testing effect explicitly calls attention to the importance of the similarity between the initial and final testing conditions. This theory suggests that testing effect derives from the overlap in material processing that occurs during the initial and final testing [19]. However, some results clearly challenge this theory: Carpenter & DeLosh (2006) fully crossed both the initial and final test types (free recall, cued recall, and recognition) and found that regardless of the format of the final test, the free recall initial testing yielded the best performance [49]. Further studies with similar methodology in patients with schizophrenia would shed light on the impact of practice effect in RP. In addition, Goldberg et al. showed a gain of 0.36 in effect size (Cohen’s d) upon repeated neuropsychological testing in first-episode psychosis patients treated with second-generation antipsychotics, while in our sample of patients, RP caused a gain of 1.04 in effect size in comparison to restudy. Though our results do not apply to global cognition, and though the comparison of effect-sizes must lead to careful conclusions, they suggest that it is quite unlikely that the superiority of RP over restudy at a later cued recall only stems from a practice effect.

Our study shows that in the retrieval practice condition, the items with weak cue-to-target association were better recalled than the items with no semantic association (in both the patient and control groups). These results are consistent with previous findings in healthy populations and clinical populations with memory deficits, where RP was regularly shown as fostering better final recall for weakly associated semantic items compared to strongly or non-associated items [19, 22]. Contrary to our findings, when using RP in patients with schizophrenia, previous studies have shown that patients did not improve their performance at final recall when weak categories-exemplar pairs were used. For example, using a RIF paradigm, Allen et al. showed that patients recalled as many weak category-exemplar pairs in the RP+ conditions as in the NRP conditions (36.7% in both conditions), while the patients improved their performance with RP for strong categories-exemplar pairs (61.7% recalled in the RP+ condition vs. 45% in the NRP condition) [27]. These results may be explained by the significant differences between our procedures and those of Allen et al. First, feedback was not provided in the previous studies (that is, unsuccessfully retrieved items were not presented again during the initial test). However, the meta-analysis by Rowland et al. clearly showed that feedback has a substantial effect on the final memory test scores (Hedge’s g = 0.39 without feedback vs. 0.73 with feedback) [19]. Moreover, in studies without feedback, a reliable testing effect of medium effect size (Hedge’s g = 0.56) was found when the initial retrieval success was greater than 75%. In contrast, when the initial test performances were scored between 51 and 75%, a testing effect of small effect size was yielded (Hedge’s g = 0.29) [19]. Such differences in the initial cued recall were observed by AhnAllen et al.: scores at the initial cued recall for strong and weak associates were 92 and 82%, respectively, in controls, and 80 and 53%, respectively, in patients [29]. Actually, as scores at the initial cued recall for weak associates were low in the patients with schizophrenia, the absence of feedback may have prevented the favourable effect of RP in this condition. These results, compared to ours, confirmed that feedback improves cued recall in patients with schizophrenia as much as in the control participants. In addition, studies of healthy subjects receiving feedback with low initial test performances (≤ 50%) yielded larger effect sizes (g = 0.99) than those with moderate (51–75%; g = 0.68) and high (> 75%; g = 0.40) initial test performances [19]. In our study, as patients demonstrated lower initial performances than controls (43.4% vs. 56.5% of words recalled at the initial phase respectively), feedback may have fostered the occurrence of a testing effect in both our samples. Secondly, retention delays in studies based on RIF procedure were short (5–20 min) and may have prevented the occurrence of a testing effect [27–30]. Instead, our protocol set a retention interval at 2 days, based on previous findings indicating that longer retention intervals (≥ 1 day) yielded larger testing effects. As testing-effect appears to grow with the duration of the retention interval and persists with very long retention intervals in healthy subjects (at least several months) [19, 26], demonstrating a similar pattern in patients with schizophrenia would confirm its interest for cognitive remediation.

Our results also show that patients’ scores at the final cued-recall test did not reach the baseline performance of the healthy controls. These findings might be explained by the theory of generalised deficit in schizophrenia [50], and they align with previous research investigating practice effect in these patients. For example, Goldberg and collaborators indicated that despite the improvement due to repeated testing, patients started and ended lower than the controls [51]. However, if we base our hypotheses on the generalised deficit theory, we would also expect a lower improvement using RP in the patients with schizophrenia in comparison to the controls. On the contrary, we found that the patients benefitted from RP as much as healthy controls (the group by condition interaction was not relevant), supporting the idea that the cognitive mechanisms underlying testing effect are preserved in patients with schizophrenia. This interpretation is further supported by the semantic association effect in our study. We know from the literature regarding semantic memory that the patients’ impairment is due to difficulties in self-initiating effective semantic encoding strategies, and that the patients’ performances can improve or even normalise when effective strategies are explicitly given to them [52–54]. Yet, our study did not use explicit instructions, but may have rather stimulated a specific encoding process (RP) that implicitly produced semantic encoding strategies. This interpretation is consistent with a previous study that concluded that the reduced processing of semantic relationships during encoding is less explained by the implicit activation and retrieval of the patients’ semantic lexicon than by the reduced ability for the patients to implement explicit relational processing strategies [53]. However, further studies using RP with explicit in comparison to implicit instructions are needed to confirm this hypothesis using a similar procedure as ours. Apart from this, as a family of theories (namely retrieval effort theories) supposes that testing effect relies on both the quality and intensity of processing that is induced by the initial retrieval attempt, this ability to process semantic information may be maintained, though untrained in patients with schizophrenia. For instance, Bjork and Bjork assume that difficult tests foster storage strength of a memory (i.e., the memory is durably established to a greater degree), thus leading to effective memory retrieval. Support for this family of theories comes from the studies showing that difficult initial tests and increased delay between the initial study and the initial test both produce larger testing effect. However, further studies manipulating the difficulty of the task are needed to examine whether the retrieval practice effect observed in our sample of patients can be explained by this effect.

Our interpretation of the results underscores a preserved ability to retrieve during the final test the existing semantic associations which were encoded at study. We have to acknowledge that this statement may be at odds with the previous literature showing that associative or relational memory processes are especially impaired in schizophrenia [55]. In our study, we chose to compare weakly and non-associated pairs as our aim was to demonstrate that RP could be a relevant cognitive therapy tool and as we considered non-associated word pairs being of greater interest in a cognitive remediation perspective (items to learn in daily life are in fact often unrelated: e.g., name and address, shopping list, password and login). However, as RP was regularly shown as fostering a greater testing effect for weakly associated semantic items in comparison to strongly-associated items in healthy populations, our hypotheses might be challenged in a further study comparing strongly and weakly associated word pairs. A similar result in patients would support the idea of a preserved ability to retrieve previously encoded semantic associations in schizophrenia, whereas other patterns of results (a stronger testing effect with the strongly associated pairs, or no difference between the strongly and weakly associated word pairs) would confirm semantic memory impairments in schizophrenia. The elaborative retrieval hypothesis, developed by Carpenter, tries to explain this greater improvement in memory recall with the weakly-associated pairs in comparison to the strongly associated pairs. This theory considers that engaging in a retrieval attempt produces semantic associations with the target, thus effectively guiding its retrieval (e.g., with the cue-target pair “PINEAPPLE-ISLAND”, a participant may generate plausible but incorrect candidates [e.g., fruit, exotic, etc.] before reaching the target [island]) [56]. As the strongly associated items require little elaboration to be retrieved, restudying these items generates some accurate answers in itself. In contrast, restudying weakly associated items, which require much elaboration to be retrieved, does not activate effortful semantic elaboration and is less efficient than RP, the latter fostering semantic associations. Following this theory, our results suggest that patients with schizophrenia are not only able to retrieve previously learned material through basic rote repetition, but also to elaborate semantic associations at an initial test and to retrieve these associations at a final cued-recall. Moreover, this ability was not affected by the neurocognitive dysfunction revealed during the initial neuropsychological examination of our patients.

Some limitations of our study must be acknowledged. First, our sample size was small, yet similar to sample sizes of previous studies on retrieval practice performed in clinical populations with memory deficits (which included between 10 and 73 participants) [21, 24] and in patients with schizophrenia (between 10 and 30 participants) [27–30]. Moreover, the Bayesian analysis method we used made it possible to test whether our data are sensitive to priors that may counter our conclusions. The analysis clearly showed that the results remained unchanged with non-informative priors and with pessimistic priors minoring possible effects. Secondly, though the patients recalled significantly more words at the final cued-recall test in the RP condition, the generalisation of benefits in episodic memory to other memory tasks remains to be demonstrated. A protocol including various cognitive tasks in verbal and/or visual episodic memory, before and after RP, might resolve this issue [57]. It is worth stressing here that although the patients exhibited poorer cognitive performance during our neuropsychological assessments, taking global cognitive performance into account did not influence the effect of retrieval practice or the interaction between group and retrieval practice, suggesting that retrieval practice might benefit patients whatever their level of cognitive deficits.

We think that our findings open new perspectives in cognitive remediation strategies, which are a pillar of recovery, in schizophrenia. RP has been previously considered as a compensatory approach to memory rehabilitation in clinical populations with memory deficits [20, 21]. In restorative approaches, RP could be integrated into computerised CT programs to train episodic memory. Indeed, as Carpenter showed evidence for testing effect transferability in terms of temporal contexts, test formats and knowledge domains, this training might foster the use of RP by patients in daily life situations [58]. However, though most cognitive remediation techniques to date rely on a computerised drill-and-practice approach alone, they appear to yield stronger effect sizes when strategy coaching is added [5, 59]. Thus, RP could also be taught in compensatory cognitive training among mnemonic strategies for remembering new information. For example, patients may engage in self-testing when they need to remember associated items in a shopping list (e.g., cheese-bread) or non-associated items (e.g., the name of a person with a photo or an address). RP might also be an effective complementary strategy to improve patients’ medication management, as episodic memory deficits predict patients’ lower medication adherence [60]. For example, patients’ relatives or health care providers may foster RP in patients by testing them about their next appointment date or their medication regimen, rather than repeating it twice or more. Furthermore, the results reported by previous studies on CT showed a great heterogeneity, depending on the patients and cognitive remediation programs [61, 62]. In our study, as RP was found effective in every patient but one, it appears to be a promising method of cognitive remediation. However, since the patients’ final cued recall scores using RP did not reach the baseline performance of the healthy controls, further investigations are necessary in order to explore the factors which might contribute to robustly improve patients’ performance while using RP. Then, future studies on cognitive remediation programs that include RP might show how long-term RP training can improve episodic memory and the quality of life in patients with schizophrenia.

## Conclusion

Retrieval practice is an efficient long-term episodic memory strategy in healthy subjects. Our study is the first to demonstrate that retrieval practice is also superior to restudy in improving later recall in patients with schizophrenia presenting with episodic memory impairment. While semantic relational memory deficits in schizophrenia mostly rely on a difficulty to self-initiate semantic memory strategies, RP may foster semantic elaboration, leading to memory performance enhancement. As memory impairment severely affects patients’ daily lives, retrieval practice, integrated into cognitive remediation programs, should be considered as a valuable tool to address these deficits and to help reduce disability. Further investigations are needed to explore the factors that promote the effectiveness of RP in patients in the context of cognitive remediation.

## Additional file

**Additional file 1: Table S1.** Estimation of the informative priors. **Table S2.** Percentage of words recalled across condition in the initial and final memory test in patients with schizophrenia and controls. **Table S3.** Results of the multivariate analyses. **Table S4.** Results of the multivariate analyses using frequentist statistical analyses (ANOVA). **Table S5.** Results of the sensitivity analyses using non-informative and pessimistic priors.

## Data Availability

The datasets generated and analysed during the current study are not publicly available due to no permission from participants to share anonymized participant data publicly but are available from the corresponding author on reasonable request.

## Abbreviations

CDSS: Calgary Depression Scale for Schizophrenia
CT: Cognitive Training
PANSS: Positive And Negative Syndrome Scale
RP: Retrieval Practice
RS: Restudy
SNRI: Serotonin and Norepinephrine Reuptake Inhibitor
SSRI: Selective Serotonin Reuptake Inhibitor

## Footnotes

1 see to that end a special issue on this topic published in *Psychonomic Bulletin & Review* in February 2018

2 Each OR corresponds here to exp.(Theta) where Theta is the coefficient of the tested factor in the Beta regression.
