# Supplementary material for: Retrieval practice improves memory in patients with schizophrenia: new perspectives for cognitive remediation
Source: BMC Psychiatry. 2019 Nov 11;19:355. doi: 10.1186/s12888-019-2341-y (PMC6849190; doi:10.1186/s12888-019-2341-y)
Supplement: Supplementary file 1 — Additional file 1: Table S1. Estimation of the informative priors. Table S2. Percentage of words recalled across condition in the initial and final memory test in patients with schizophrenia and controls. Table S3. Results of the multivariate analyses. Table S4. Results of the multivariate analyses using frequentist statistical analyses (ANOVA). Table S5. Results of the sensitivity analyses using non-informative and pessimistic priors. [file 12888_2019_2341_MOESM1_ESM.docx]

Table S1. Estimation of the informative priors**.**

The means and SD for condition and semantic association from Akdogan et al. (2016) are presented in the left part of the Table below. Informative priors (i.e. estimated entered in the Beta regression) were calculated using the formulas below and are reported in the right part of the table.

For testing

- M_e_alpha was calculated using log( (M_p_restudy /(1-M_p_restudy) )
- SD_e_alpha was calculated using (CI_e_alpha 97.5% - CI_e_alpha 2.5%) / (2*1.96)
- Precision_e_alpha was calculated using 1/SD_e_alpha^2
- M_e_theta was calculated using log( (M_p_restudy + M_p_testing)/(1-M_p_restudy - M_p_testing ) )
- SD_e_theta was calculated using (CI_e_theta 97.5% - CI_et_heta 2.5%) / (2*1.96)
- Precision_e_theta was calculated using 1/SD_e_theta^2

Similar procedure was used to estimate the parameters for semantic link

|  |  | **data**  **from Akdogan et al. (2016)** | | | | *parameters* | **estimated parameters**  **for the Beta regression** | | | | |
| --- | --- | --- | --- | --- | --- | --- | --- | --- | --- | --- | --- |
|  |  | *M_p* | *SD_p* | *CI_p* | |  | *M_e* | *CI_e* | | *SD_e* | *Precision_e* |
|  |  |  |  | 2.5% | 97.5% |  |  | 2.5% | 97.5% |  |  |
| condition | restudy | 0.570 | 0.162 | 0.252 | 0.888 | **alpha** | **0.282** | -1.085 | 2.066 | 0.804 | **1.548** |
|  | testing | 0.694 | 0.180 | 0.341 | 1.047 |  | 0.819 | -0.658 | 3.108 | 0.961 | 1.084 |
|  | difference | 0.128 | 0.184 | -0.233 | 0.489 | **theta** | **0.556** | -0.226 | 1.730 | 0.499 | **4.018** |
|  |  |  |  |  |  |  |  |  |  |  |  |
| semantic association | weak | 0.486 | 0.228 | 0.039 | 0.933 | **alpha** | -**0.056** | -3.201 | 2.632 | 1.488 | **0.452** |
|  | strong | 0.758 | 0.153 | 0.458 | 1.058 |  | 1.142 | -0.168 | 2.906 | 0.784 | 1.627 |
|  | difference | 0.275 | 0.146 | -0.011 | 0.561 | **theta** | **1.214** | 0.524 | 2.334 | 0.462 | **4.695** |
|  | *Note*. M_p = Mean of the known parameter; SD_p = standard deviation of the known parameter; CI_p = Credible Interval of the known parameter; M_e = Mean of the estimated parameter; SD_e = standard deviation of the estimated parameter; CI_e = Credible Interval of the estimated parameter; Precision_e = precision of the estimated parameter (precision = 1/SD^2) | | | | | | | | | | |

These priors were then entered in the Beta regression as follows (jags model in R used in our analyses):

model<-function(){

for (i in 1:N){

numberofpairs[i]~dbeta(a[i], b[i])

a[i]<- mu[i]*gamma

b[i]<- (1-mu[i])*gamma

logit (mu[i]) <- alpha + theta[predictor[i]] + priorsuj[suj[i]]

}

for(n in 1:NSuj) {

priorsuj[n]~ dnorm(0,tau) }

tau~dgamma(0.1,0.1)

alpha~dnorm(**M_e_alpha,Precision_e_alpha**)

theta[1]<-0

theta[2]~dnorm(**M_e_theta,Precision_e_theta**)

OR<-exp(theta[2])

PrOR<-step(OR-1)

gamma~dgamma(0.01,0.01)

}

**Table S2. Percentage of words recalled across condition in the initial and final memory test in patients with schizophrenia and controls.**

|  |  | Controls | | | Patients with schizophrenia | | |
| --- | --- | --- | --- | --- | --- | --- | --- |
|  |  | (*n* = 20) | | | (*n* = 19) | | |
|  |  | *%* | *SD* | *%* | | *SD* |  |
| **Performance at initial test** | | | | | | |  |
|  | (weak+no) | 56.5 | 15.5 | 43.4 | | 17.4 |  |
| **Performance at final test** | | | | | | |  |
| Test | Weak association | 85.3 | 16.7 | 67.3 | | 26.0 |  |
|  | No association | 42.7 | 32.0 | 28.7 | | 18.7 |  |
| Restudy | Weak association | 57.3 | 24.7 | 34.7 | | 27.3 |  |
|  | No association | 27.3 | 26.7 | 12.0 | | 14.0 |  |
| Test | (weak+no) | 63.8­­ | 32.8 | 47.0 | | 30.1 |  |
| Restudy | (weak+no) | 42.3 | 29.7 | 27.7 | | 25.8 |  |
| Weak association | (restudy+test) | 71.2 | 25.1 | 57.0 | | 27.2 |  |
| No association | (restudy+test) | 35.0 | 29.9 | 17.7 | | 15.2 |  |
| All conditions | | 53.1 | 32.9 | 37.4 | | 29.5 |  |

**Table S3. results of the multivariate analyses.**

|  | *M* | *SD* | *CI* | |  |
| --- | --- | --- | --- | --- | --- |
|  |  |  | *2.5%* | *97.5%* | *Pr(OR>1)* |
| alpha | -1.178 | 0.292 | -1.762 | -0.612 |  |
| OR_group | 0.387 | 0.189 | 0.177 | 0.896 | 0.013 |
| OR_semantic | 4.724 | 0.932 | 3.257 | 6.904 | 1.000 |
| OR_condition | 2.347 | 0.436 | 1.648 | 3.350 | 1.000 |
| OR_Inter_group_semantic | 1.334 | 0.391 | 0.776 | 2.296 | 0.852 |
| OR_Inter_group_condition | 0.988 | 0.296 | 0.570 | 1.720 | 0.483 |
| OR_Inter_semantic_condition | 2.219 | 0.638 | 1.306 | 3.793 | 0.998 |
| OR_Inter_group_semantic_condition | 0.759 | 0.336 | 0.353 | 1.641 | 0.241 |

**Table S4. Results of the multivariate analyses using frequentist statistical analyses (ANOVA).**

|  | F | p | Partial η^2^ |
| --- | --- | --- | --- |
| Group | 5.287 | .027* | .128 |
| Condition | 119.1 | <.0001* | .768 |
| Semantic relatedness | 240.6 | <.0001* | .870 |
| Group*condition | .360 | .552 | .010 |
| Group*semantic relatedness | .405 | .529 | .011 |
| Condition*semantic relatedness | 15.8 | <.0001* | .305 |
| Group*condition*semantic relatedness | .405 | .528 | .011 |

**Table S5. results of the sensitivity analyses using non-informative and pessimistic priors.**

As mentioned above, informative priors were the following:

alpha~dnorm(**M_e_alpha,Precision_e_alpha**)

theta[2]~dnorm(**M_e_theta,Precision_e_theta**)

Non-informative priors were:

alpha~dnorm(**0, 0.01**)

theta[2]~dnorm(**0, 0.4**)

Pessimistic priors were:

alpha~dnorm(**M_e_alpha,Precision_e_alpha**)

theta[2]~dnorm(**- M_e_theta,Precision_e_theta**)

In other words, the same magnitude of the expected effect was introduced for an effect going in the opposite direction of our expectation.

| Priors | Semantic association | | | | |  | | Condition | | | | |  |
| --- | --- | --- | --- | --- | --- | --- | --- | --- | --- | --- | --- | --- | --- |
|  |  | *M* | *SD* | *CI2.5%* | *CI97.5%* | *Pr(OR>1)* | *M* | | *SD* | *CI2.5%* | *CI97.5%* | *Pr(OR>1)* | |
| non | theta | 1.695 | 0.141 | 1.415 | 1.967 |  | 0.827 | | 0.158 | 0.515 | 1.137 |  | |
| informative | OR | 5.453 | 0.774 | 4.118 | 7.151 | >0.999 | 2.289 | | 0.369 | 1.673 | 3.119 | >0.999 | |
| informative | theta | 1.733 | 0.147 | 1.443 | 2.017 |  | 0.865 | | 0.167 | 0.535 | 1.193 |  | |
|  | OR | 5.665 | 0.841 | 4.232 | 7.513 | >0.999 | 2.377 | | 0.405 | 1.707 | 3.297 | >0.999 | |
| pessimistic | theta | 1.457 | 0.149 | 1.159 | 1.742 |  | 0.713 | | 0.161 | 0.395 | 1.025 |  | |
|  | OR | 4.304 | 0.647 | 3.188 | 5.710 | >0.999 | 2.041 | | 0.333 | 1.485 | 2.787 | >0.999 | |

*Note*. OR = exp(Theta)
